# Reconciling Ligase Ribozyme Activity with Fatty Acid Vesicle Stability

**DOI:** 10.3390/life4040929

**Published:** 2014-12-11

**Authors:** Fabrizio Anella, Christophe Danelon

**Affiliations:** Department of Bionanoscience, Kavli Institute of Nanoscience, Delft University of Technology, Lorentzweg 1, 2628 CJ, Delft, The Netherlands; E-Mail: f.m.anella@tudelft.nl

**Keywords:** catalytic RNA, fatty acid, lipid vesicle, protocell, RNA degradation

## Abstract

The “RNA world” and the “Lipid world” theories for the origin of cellular life are often considered incompatible due to the differences in the environmental conditions at which they can emerge. One obstacle resides in the conflicting requirements for divalent metal ions, in particular Mg^2+^, with respect to optimal ribozyme activity, fatty acid vesicle stability and protection against RNA strand cleavage. Here, we report on the activity of a short L1 ligase ribozyme in the presence of myristoleic acid (MA) vesicles at varying concentrations of Mg^2+^. The ligation rate is significantly lower at low-Mg^2+^ conditions. However, the loss of activity is overcompensated by the increased stability of RNA leading to a larger amount of intact ligated substrate after long reaction periods. Combining RNA ligation assays with fatty acid vesicles we found that MA vesicles made of 5 mM amphiphile are stable and do not impair ligase ribozyme activity in the presence of approximately 2 mM Mg^2+^. These results provide a scenario in which catalytic RNA and primordial membrane assembly can coexist in the same environment.

## 1. Introduction

An RNA-based protocell, comprised of catalytic RNA molecules, called ribozymes, encapsulated inside fatty acid vesicles, has been conjectured to represent a key intermediate in the evolutionary pathway that led to the emergence of cellular life [1,2,3]. This hypothesis is supported by a number of recent breakthroughs regarding vesicle compartment growth and division [4,5,6], and self-replicating RNA [7,8], each component being examined separately. However, a scenario in which fatty acid vesicles and ribozymes coexisted can only be viable if both systems are able to fulfill their functions in the same prebiotically plausible aqueous medium. The challenge to find such unifying environmental conditions is greatly exemplified by the conflicting roles played by divalent metal ions, in particular Mg^2+^. By promoting RNA folding and catalysis [9,10], Mg^2+^ acts as a central element in the function of ribozymes and in nonenzymatic RNA copying [11]. Most ribozymes own high-Mg^2+^ concentration requirements and enzyme-free template-directed RNA synthesis usually occurs at >50 mM Mg^2+^. However, Mg^2+^ can also assume deleterious functions, namely the catalysis of RNA cleavage and the destabilization of fatty acid vesicles [12].

To remedy these problems a number of solutions have been envisaged. First, the susceptibility of fatty acid vesicles for Mg^2+^-dependent rupture can be attenuated by complementing fatty acid membranes with their corresponding alcohols and glycerol monoesters [13,14,15]. Second, the Mg^2+^-chelating agent citrate, by partially coordinating the Mg^2+^ ion, is capable to impressively increase the tolerance of fatty acid vesicles for Mg^2+^ concentration, while protecting RNA from hydrolytic degradation and retaining its catalytic role in nonenzymatic template-directed RNA polymerization [16]. Alternatively, the substitution of Mg^2+^ with Fe^2+^ (or other divalent cations) has also been proposed. In the case of some ribozymes the new metal could preserve, even enhance activity [17].

**Figure 1:**
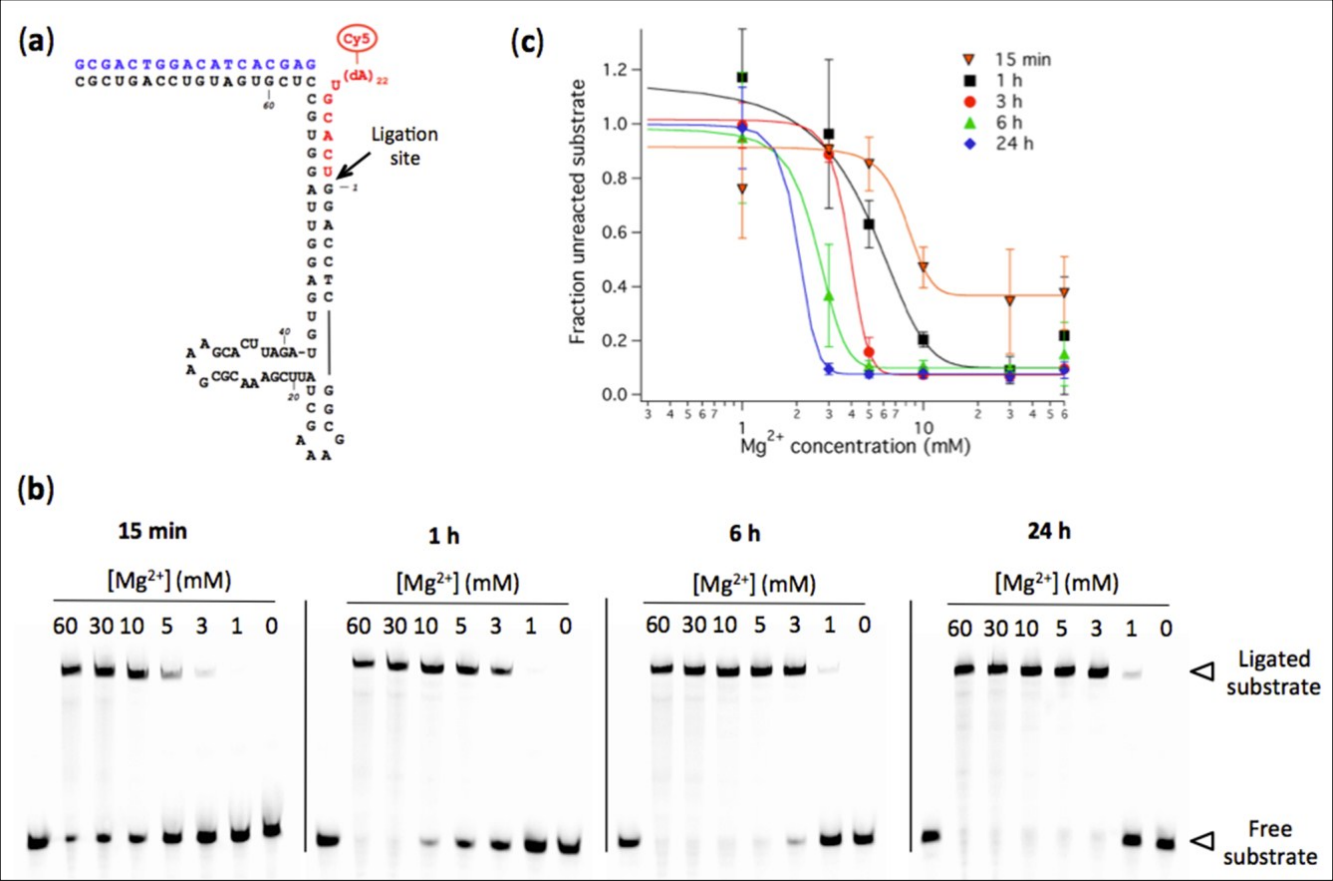
(**a**) Predicted secondary structure of the R8-9 L1 ligase. The body ribozyme is colored black, the effector oligonucleotide is in blue, and the substrate oligonucleotide conjugated to a poly(dA) tail terminated by a Cy5 dye is in red. The RNA ligase catalyzes the formation of a phosphodiester bond between the substrate and the body ribozyme as depicted by the arrow; (**b**) Polyacrylamide gel electrophoresis analysis of R8-9 ribozyme-catalyzed substrate ligation performed at the indicated Mg^2+^ concentrations and reaction times. [Ribozyme] = 1 µM, [Effector] = 2 µM, [Substrate] = 0.25 µM. The reaction buffer used was 200 mM NaCl, 30 mM Tris-HCl, 1 mM EDTA, 0.1% NP40, pH 7.7. The first lane of each gel corresponds to a substrate-only solution and the band intensity was used as the total amount of substrate *S*_0_ for normalization; (**c**) Quantitation of ligation efficiency expressed as the fraction of unreacted substrate derived from PAGE analysis. The solid lines indicate sigmoidal curve fits. Two to three independent experiments were performed for each condition; error bars represent standard deviations.

As a complementary approach to the above studies, we report here on the Mg^2+^-dependence of the L1 ligase ribozyme activity in the presence of stable myristoleic acid (MA) vesicles. The L1 RNA ligase catalyzes the formation of a phosphodiester bond between the 3’-terminal residue of a substrate oligonucleotide and the 5’-terminal guanosine triphosphate of the ribozyme [18] (Figure 1a). The L1 ligase works optimally at high Mg^2+^ concentration (around 60 mM) and is highly specific for Mg^2+^ [19]. Ligase ribozymes are particularly interested because they can potentially catalyze the condensation of short RNA fragments into longer structurally or catalytically active molecules. In this study, we used the R8-9 ligase that was selected for its short sequence and improved activity [20], and examined its tolerance for low-Mg^2+^ conditions in a high ionic strength aqueous solution. Besides, we chose MA as a reasonable prebiotic amphiphile because related fatty acids can be abiotically synthesized by Fischer-Tropsch reaction [21] and have been detected in carbonaceous meteorites [22,23]. Furthermore, MA vesicles are capable of growth and division [5] and they can encapsulate functional molecules [14].

With one notable exception [14], catalytically active RNA and fatty acid vesicle properties have been investigated separately, *i.e.*, not in the same batch of reaction. Therefore, the present study, by showing that the L1 RNA ligase retains activity in conditions compatible with MA vesicle self-assembly and integrity provides new insights on how to reconcile the “RNA world” [24] and “Lipid world” [25] hypotheses.

## 2. Experimental Section

### 2.1. Materials

All oligonucleotides were synthesized by Ella Biotech (Martinsried, Germany). Myristoleic acid (MA, C14:1) was purchased from Sigma-Aldrich Chemie B.V. (Zwijndrecht, The Netherlands) (≥99% capillary GC).

### 2.2. Preparation of the Ribozyme RNA

The DNA template encoding for the R8-9 variant of the L1 ligase ribozyme (74 ribonucleotides) [20] was obtained by polymerase chain reaction (PCR). Briefly, 0.2 µM of template (5’-TTCTAATACGACTCACTATA*GGACCTCGGCGAAAGCTATTCGAAACGCGAAAGCACTTAGATGTGAGGTTAGGTGCCTCGTGATGTCCAGTCGC*-3’; the underlined sequence is the T7 promoter sequence, the ribozyme sequence is denoted in italic) were amplified using 0.2 µM of forward primer (5’-TCCTAATACGACTCACTATA-3’) and reverse primer (5’-GCGACTGGACATCACGAG-3’) in a 50-µL reaction solution containing 200 µM of each dNTPs, 1× Phusion HF buffer, and 1 U Phusion DNA Polymerase (Thermo Scientific). The following temperature cycling conditions were used: 98 ºC for 5 s, 50 ºC for 15 s and 72 ºC for 15 s for a total of 25 cycles. The PCR product was analyzed using 2% agarose gel electrophoresis (100 W, 40 min), purified using the PCR Clean-up kit from Promega, and the amount and purity of amplified DNA were assessed by absorbance using a Nanodrop 2000c (Thermo Fisher Scientific, Waltham, MA, USA).

The L1 ligase RNA was transcribed from the purified template DNA using the RiboMAX Large Scale RNA Production System (Promega Benelux B.V., Leiden, The Netherlands). *In vitro* transcription was performed at 37 ºC overnight in a 100-µL solution containing 500 ng of template DNA, 7.5 mM NTPs and 10 µL of the T7 RNA polymerase mixture. The reaction was stopped on ice and the DNA template was degraded by DNaseI for 15 min at 37 ºC. The produced RNA was purified using the RNeasy MinElute Clean up kit (QIAGEN), and its concentration and purity were determined by absorbance (NanoDrop 2000c). The integrity of the RNA was validated by 7 M urea denaturing 12% polyacrylamide gel electrophoresis (PAGE) (120 W, 100 min) after ethidium bromide staining for 15 min.

### 2.3. Ribozyme Activity Assay in Absence of Vesicles

The purified ribozyme (1 µM) was mixed in water with two equivalents of the reverse DNA primer (used as oligonucleotide effector) and the nucleic acids were denatured by heating at 70 ºC for 5 min. Annealing was performed by cooling down the solution to room temperature and the ligation buffer (30 mM Tris-HCl, pH 7.7, 200 mM NaCl, 1 mM EDTA, 0.1% NP40 and the appropriate concentration of MgCl_2_) was added. The oligonucleotide substrate (5’-Cy5-AAAAAAAAAAAAAAAAAAAAAAUGCACU-3’) consisted of the canonical 6-ribonucleotide sequence (underlined) pairing with the L1 ligase template extended with a poly(dA) tail and fluorescently labeled at the 5’ end with Cy5. The substrate was added to a final concentration of 250 nM to begin the ligation reaction and the solution was incubated at room temperature. Aliquots of 10 µL were removed at different time points and diluted in one equivalent volume of loading buffer (0.01% bromophenol blue, 13% ficoll (w/v), 7 M urea, 2 mM EDTA, 90 mM Tris-borate; ssRNA Ladder Loading Dye, New England Biolabs) to terminate the reaction. Samples were denatured for 5 min at 70 ºC, and the unreacted RNA substrate and ligation product were separated by denaturing PAGE, and resolved by fluorescence imaging using a Typhoon Trio (GE Healthcare, Pittsburgh, PA, USA).

### 2.4. Vesicle Preparation

Fatty acid micelles were prepared by dispersing MA amphiphiles to a final concentration of 80 mM in milliQ water containing 90 mM of NaOH (pH 13) final. The solution was then vortexed briefly and tumbled overnight at room temperature. Argon was continuously flushed in the solution for 30 min before use in order to ensure complete dissolution of any remaining oil droplets. Vesicles were formed by adding the micelle stock solution to different volumes of 200 mM sodium bicine buffer, pH 8.5, or to the ligation buffer modified as follows: 30 mM Tris-HCl, pH 7.7, 100 mM NaCl, 1 mM EDTA, 2 mM MgCl_2_. When indicated, 1 µM of ribozyme was supplemented in the ligation buffer prior micelle addition. In some experiments, vesicles were extruded through a 200-nm polycarbonate membrane using an Avanti mini-extruder and the effector-ribozyme complex was supplemented to the preformed vesicles.

### 2.5. Vesicle Characterization by Turbidity Change and Dynamic Light Scattering

The kinetics of MA vesicle formation was monitored based on turbidity change upon micelle dilution (5 to 25 mM lipid concentrations final) to the modified ligation buffer (supplemented or not with MgCl_2_ or ribozyme) by measuring the absorbance at 400 nm at room temperature as a function of time in a microplate reader (Wallac 1420 Victor2, Perkin Elmer, Waltham, MA, USA) using a 384-well plate (Small Volume™ LoBase Polystyrene Microplates clear, Greiner Bio-One B.V., Alphen a/d Rijn, The Netherlands). A blank solution consisting of the micelle buffer was used and the results are displayed as absorbance changes. Note that we use both terms “absorbance” and “turbidity” changes to define the change in light scattering signal induced by vesicles or larger aggregates assembly.

To determine vesicle integrity and size distribution, dynamic light scattering (DLS) measurements were carried out on a Zetasizer Nano ZS (Malvern, UK) operating at a scattering angle of 173º at room temperature. The vesicle solution (5 to 25 mM lipid concentrations final) was equilibrated for at least 4 h at room temperature and a 70-µL sample was added into a disposable microcuvette (ZEN0040, Malvern). For each condition, at least two independent biological samples were analyzed and three runs of 60–70 s duration were generated for each sample. An attenuation factor of 5 to 10 was used. The DLS data are represented as weighted intensity distribution *versus* apparent hydrodynamic diameter and a representative curve is shown for each condition. It is worth noting that in DLS sizing big particles dominate the light scattering signal and are over-represented in the weighted intensity profiles. The raw correlation data, weighted volume distributions and polydispersity indices were also inspected to help interpret the results and estimate the confidence in the reliability of the data.

### 2.6. Ribozyme Activity Assay in Presence of Vesicles

The L1 ribozyme (transcription product, 1 µM final concentration) was pre-annealed with 2 µM (final concentration) of effector as described in Section 2.3 and the complex was added to a vesicle solution consisting of 5–25 mM MA amphiphile in the modified ligation buffer (30 mM Tris-HCl, pH 7.7, 100 mM NaCl, 1 mM EDTA, 2 mM MgCl_2_). After the solution was equilibrated for 2 h, the ligation reaction was initiated by adding 0.25 µM (final concentration) of Cy5-conjugated substrate. Ligation stop, sample denaturation and visualization of the reaction product were performed as described in Section 2.3.

### 2.7. Gel Analysis

**Figure 2:**
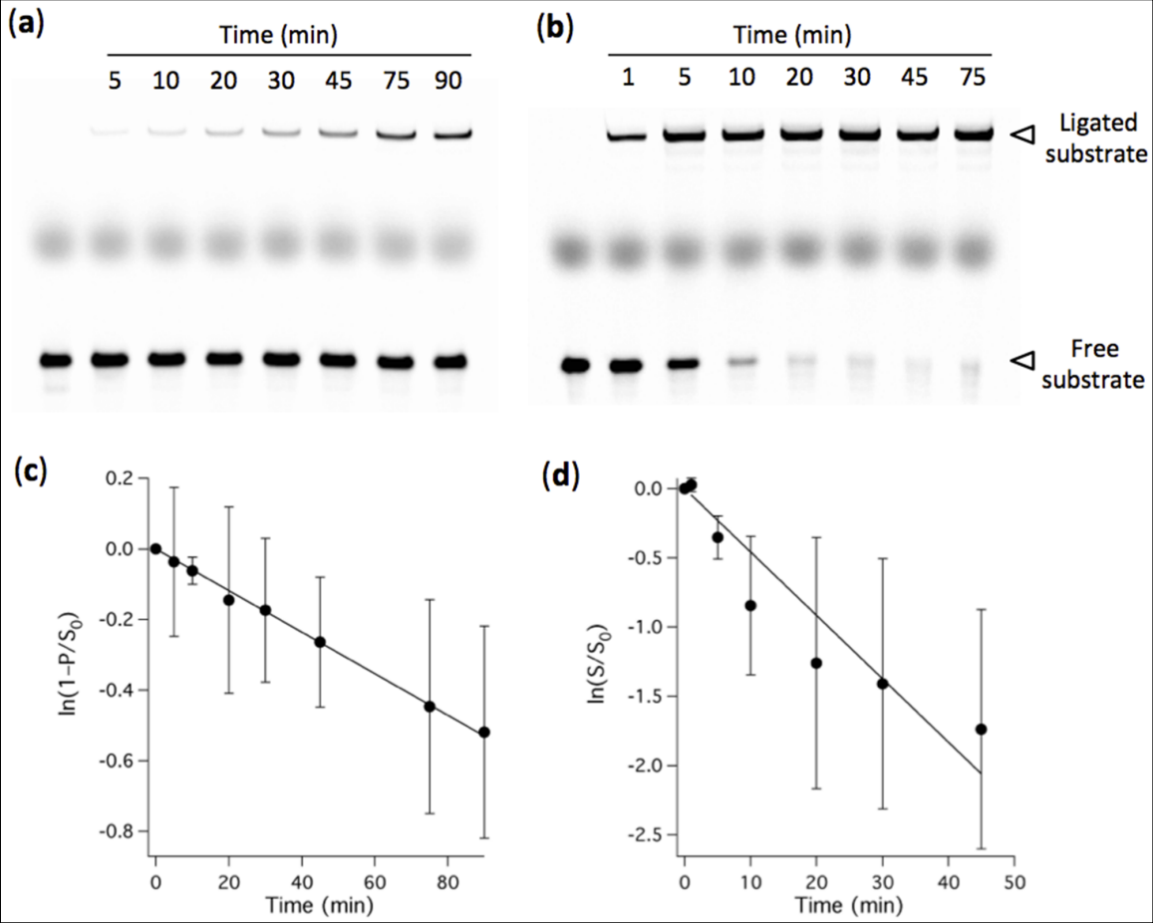
PAGE analysis of ligation kinetics performed in the presence of 3 mM Mg^2+^ (**a**) or 60 mM Mg^2+^ (**b**). [Ribozyme] = 1 µM, [Effector] = 2 µM, [Substrate] = 0.25 µM. The reaction buffer used was 200 mM NaCl, 30 mM Tris-HCl, 1 mM EDTA, 0.1% NP40, pH 7.7. The first lane of each gel corresponds to a substrate-only solution and the band intensity was used as the total amount of substrate *S*_0_ for normalization. The thick middle band corresponds to the loading dye. (c, d) Quantitative analysis of the ligation rate. (**c**) The fraction of ligated substrate under 3-mM-Mg^2+^ condition was measured. (**d**) The fraction of remaining substrate under 60-mM-Mg^2+^ condition was calculated. (c, d) Solid lines indicate linear fits whose slopes give the apparent ligation rate constants. Three independent experiments were performed for each condition; error bars represent standard deviations calculated as described in the experimental section.

PAGE analysis was performed by quantifying the band intensities using the ImageJ software [26] and the fraction of unreacted substrate (*S*/*S*_0_) was calculated at a given time point or Mg^2+^ concentration. The apparent ligation rate constant was determined by linear regression of the natural logarithm of *S*/*S*_0_ *versus* time (e.g., Figure 2d). When the decrease of the band intensity corresponding to the unreacted substrate was not pronounced enough for direct quantification, then the ratio *S*/*S*_0_ was calculated as (1 – *P*/*S*_0_), where *P*/*S*_0_ denotes the ratio of the amount of ligated substrate to that of the total substrate (Figure 2c). Error bars in Figure 2c,d were calculated as the standard deviations of the values *S*/*S*_0_ (or *P*/*S*_0_) divided by the mean values. When titrating the amount of Mg^2+^ or that of MA, the concentration corresponding to half-maximal activity was determined by fitting the fraction of unligated substrate with a sigmoidal curve.

### 2.8. Fluorescence Microscopy

Vesicles prepared from 5 mM MA in the ligation buffer devoid of NP40 and containing either 2 mM or 8 mM Mg^2+^ were labeled using 1 µM rhodamine 6G (Sigma-Aldrich) as a lipid dye. A 10-µL-solution droplet was squeezed into a homemade silicon chamber mounted onto a #1.5 glass coverslip. The sample was imaged using a fluorescence confocal microscope (A1^+^ from Nikon) equipped with a ×100 oil immersion objective, and a 561-nm laser line with appropriate dichroic mirror and emission filter.

## 3. Results and Discussion

### 3.1. Influence of Mg^2+^ Concentration on Ligase Ribozyme Activity

We first examined the influence of Mg^2+^ concentration on the ligation rate of the R8-9 ribozyme. The conversion of oligonucleotide substrate into a longer ligation product by pre-assembled ribozyme-effector complexes was measured using a fluorescence-based band-shift assay (Figure 1). The ligase ribozyme activity, expressed as the fraction of remaining (unreacted) substrate, was quantified at varying amount of Mg^2+^ and different reaction times. Decreasing Mg^2+^ concentration from 60 mM to 1 mM is accompanied by a decrease in ligase activity (Figure 1b). The concentrations of Mg^2+^ corresponding to half-maximal activity are 8.0 ± 1.7 mM (15 min), 4.6 ± 1.4 mM (1 h), 3.9 ± 0.2 mM (3 h), 2.6 ± 0.8 mM and 2.0 ± 1.7 mM (24 h). After 48 h and 72 h reaction, a small fraction of substrate could be ligated even at 1 mM Mg^2+^: 16% ± 4% after 48 h and 25% ± 12% after 72 h, respectively, corresponding to 40 nM and 62 nM of ligation product starting from 250 nM substrate (not shown on gel). The observed ligation rate constant was determined in the presence of 60 mM and 3 mM of Mg^2+^, and values of 2.8 ± 0.8 h^−1^ and 0.35 ± 0.01 h^−1^ were obtained, respectively (Figure 2).

Therefore, our results indicate that even under low-Mg^2+^ conditions (<5 mM) ribozyme-catalyzed ligation can occur, though its rate is markedly lower. The extent to which the rate of ribozyme-assisted reactions could have been limiting in the context of an “RNA world” remains to be investigated.

To assess the possible influence of Cl^−^ on the Mg^2+^-dependent ligation rate, we conducted a few ribozyme activity experiments in which Cl^−^ was substituted with acetate as a counter ion of Na^+^. We found that the yield of the ligation reaction is very similar for Cl^−^ and acetate anions over the full range of Mg^2+^ concentrations tested (not shown).

Note that the indicated Mg^2+^ concentration corresponds to the total concentration in the solution. Because the ligation buffer contains 1 mM EDTA the concentration of free Mg^2+^ available to participate to ribozyme folding and catalytic reaction is lower than the indicated value.

Through the specific example of the R8-9 ligase ribozyme, these results suggest that ribozymes with ligase activity may have lower Mg^2+^ requirements than previously expected [14]. This extends the range of ionic compositions compatible with fatty acid vesicle integrity.

### 3.2. Influence of Mg^2+^ Concentration on the Formation of MA Vesicles

Though the detrimental effect of Mg^2+^ on fatty acid vesicle stability has long been demonstrated, the precise Mg^2+^ concentration tolerated strongly depends on the global ionic composition of the aqueous medium, in particular on the concentration of monovalent inorganic ions, such as Na^+^ and Cl^−^. Therefore, we investigated the formation and stability and MA vesicles exposed to different concentrations of Mg^2+^ in the modified ribozyme reaction buffer (30 mM Tris-HCl, pH 7.7, 100 mM NaCl, 1 mM EDTA). Note that the NP40 surfactant has been omitted to avoid interference with vesicle self-assembly.

**Figure 3:**
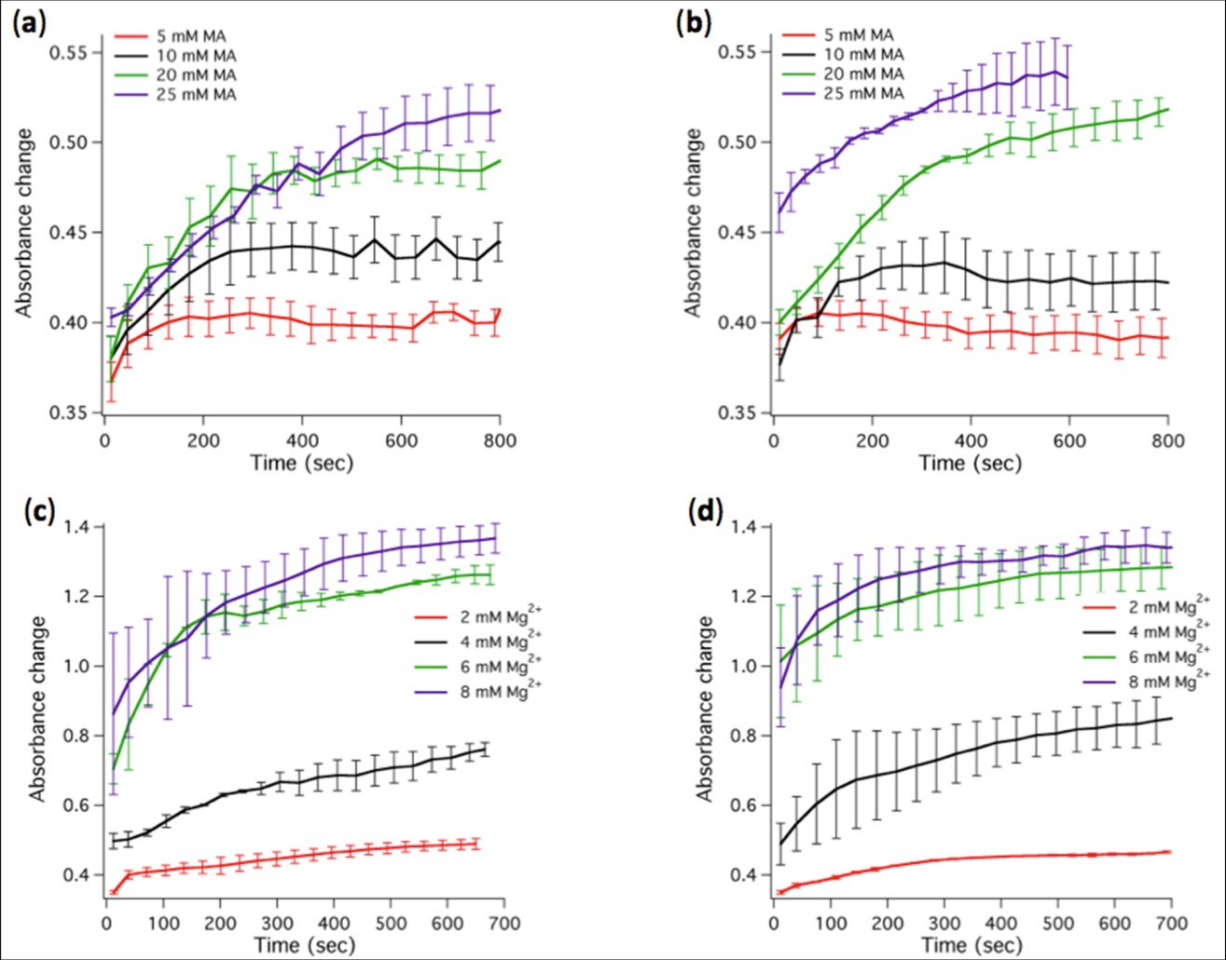
Kinetics of absorbance changes associated to micelle-vesicle transition or fatty acid precipitation. (**a**) Absorbance changes for varying MA concentrations in the modified ligation buffer containing 2 mM Mg^2+^. (**b**) Same as in (a) except that 1 µM ribozyme was present in the ligation buffer. (**c**) Absorbance changes for varying Mg^2+^ concentrations in the presence of 20 mM MA. (**d**) Same as in (c) except that 1 µM ribozyme was present. Two to three independent experiments were performed for each condition; error bars represent standard deviations. The indicated Mg^2+^ concentrations correspond to total concentrations.

The transfer of MA from micelles to vesicles was monitored as turbidity changes over time. We found that stable vesicles could self-assemble at MA concentrations between 5 mM and 25 mM in the presence of 2 mM Mg^2+^ (Figure 3a). Increasing Mg^2+^ concentration above 4 mM causes MA (20 or 25 mM) to precipitate as indicated by the large rise in absorbance (Figure 3c) and by the visible increase of the turbidity of the solution.

**Figure 4:**
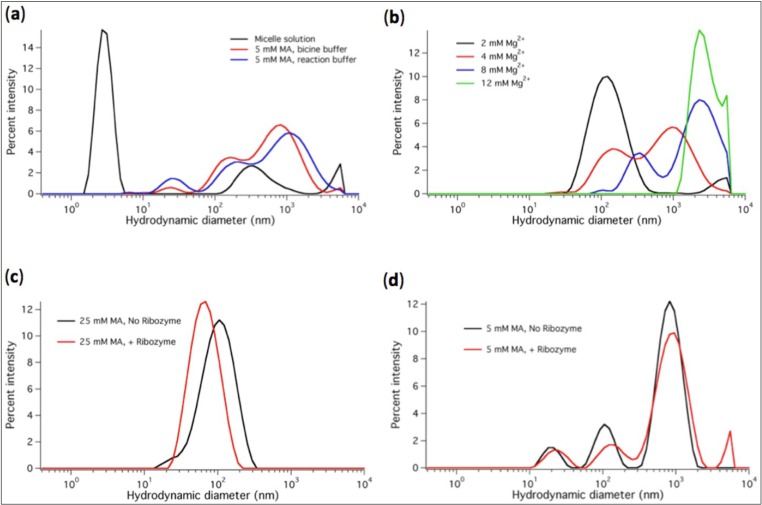
DLS measurements performed at varying conditions are displayed as percent intensity vs apparent hydrodynamic diameter. (**a**) Diameter profiles of MA micelles (80 mM MA in NaOH solution) and MA vesicles formed in the bicine buffer and ligation buffer containing 2 mM Mg^2+^. In the micelle sample, minor peaks around 300 nm and above 5 µm reflect the presence of a very small fraction of larger aggregates. However, these peaks are not observed in every measurements and the fraction of micelles is clearly prominent with a consistently low polydispersity index (<0.3) of the distribution. (**b**) Size distributions in aqueous solutions containing 20 mM MA and varying Mg^2+^ concentrations. (**c**) Vesicle diameter profiles in solutions containing 25 mM MA and 2 mM Mg^2+^, and without or with 1 µM ribozyme. (**d**) Vesicle diameter profiles in solutions containing 5 mM MA and 2 mM Mg^2+^, and without or with 1 µM ribozyme. Vesicles were formed starting from micelles diluted in the indicated buffer. For each condition a representative profile from at least two independent experiments (three runs each) is shown. Note that the differences in the curves with and without ribozyme (c, d) are within the variability range of the DLS measurements and cannot be attributed to ligase-specific effects.

**Figure 5:**
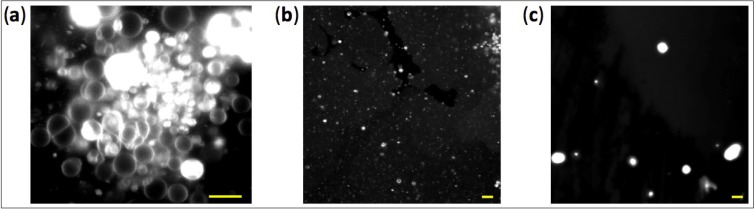
Fluorescence confocal images of MA vesicles. The vesicle solution contains 2 mM (**a**, **b**) or 8 mM (**c**) Mg^2+^. MA concentration was 5 mM. Scale bars represent 6 µm. In panel (a), the white areas are multilamellar vesicles whose fluorescence signal saturates. We chose to show a fluorescence image of a cluster of large vesicles sedimented on the glass surface to have a sufficiently large number of spatially-resolved liposomes in one field of view (a). Vesicles dispersed in the solution are also observed but their low density and motion make the imaging more complicated (b). Note that the size of vesicles below around 300 nm in diameter cannot be accurately determined due to the optical diffraction limit of the microscope.

We also studied the micelle-to-vesicle transition and Mg^2+^-dependent precipitation of liposomes using DLS. A characteristic peak centered at 3-nm hydrodynamic diameter was observed for the micelle solution (Figure 4a). The particle size distribution was shifted to diameters ranging between 0.1 and 3 µm when MA was suspended at 5 mM (or higher) concentration in bicine buffer (used as a typical medium for MA vesicle assembly) or in the modified ligase buffer containing 2 mM Mg^2+^ (Figure 4a,c,d). When Mg^2+^ reached concentrations higher than 4 mM the size distribution was further shifted to bigger particle diameters, consistent with vesicle disruption and MA precipitation (Figure 4b). Given that vesicles can agglutinate into larger scattering particles (Figure 5a), it is important to realize that the measured (apparent) hydrodynamic diameter profile does not effectively reflect the size of individual vesicles. Agglomerated vesicles contribute to the multimodal intensity distributions and leads to substantial variability even between consecutive runs on the same biological sample. Besides, DLS data are very sensitive to sample preparation, in particular when vesicles are formed from micelles. Nonetheless, the presence of either micelles, vesicles at 5 mM MA, vesicles at 20–25 mM MA or Mg^2+^-induced aggregates could unambiguously be identified and discriminated.

To have more direct insights on vesicle properties and on the effects of Mg^2+^ concentration we visualized the structures formed by 5 mM MA doped with a small fraction of the fluorescent dye rhodamine 6G using confocal microscopy. Intact spherical vesicles are clearly observed in the presence of 2 mM Mg^2+^ (Figure 5a,b), whereas larger aggregates are formed at 8 mM Mg^2+^ (Figure 5c), in agreement with both turbidity and DLS measurements.

Taken together, these data suggest that an Mg^2+^ concentration of 1 to 3 mM offers a good compromise between MA vesicle integrity and L1 ligase activity.

### 3.3. The Presence of Ligase Ribozyme Does Not Influence the Formation of MA Vesicles

Next we sought to investigate whether the presence of ligase ribozyme could influence the self-assembly of MA vesicles upon micelle dilution into the ligation buffer. Both turbidity change (Figure 3b,d) and DLS (Figure 4c,d) measurements show that addition of 1 µM of R8-9 RNA ligase has no significant effects on the kinetics of vesicle formation, stability and size distribution (Figure 3 and Figure 4). The curve shifts seen in the DLS data (Figure 4c,d) are typical run-to-run differences and thus are not ascribed to ribozyme effects. Because of the variability inherent to DLS measurements of polydisperse particle suspensions, additional experiments were carried out using preformed vesicles extruded through a 200-nm polycarbonate membrane. No effect of ribozyme on vesicle size distribution or aggregation state was observed by DLS analysis of a monodisperse population of liposomes; in both conditions (without and with 1 µM ligase) a single peak in the hydrodynamic diameter profile centered around 190 nm was measured (data not shown).

### 3.4. High Concentration of MA Vesicles Impairs Ribozyme-Catalyzed Ligation

**Figure 6:**
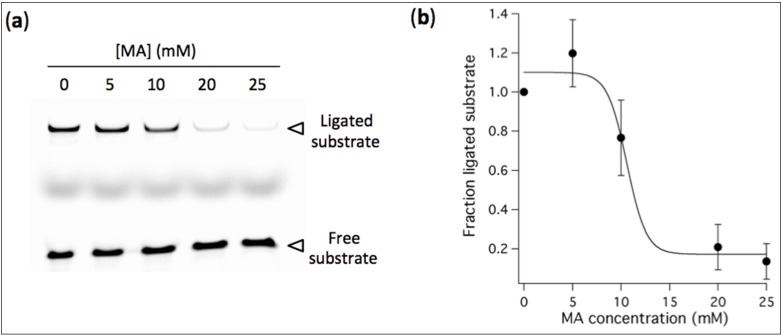
(**a**) Ligation assay performed in the presence of preformed vesicles at indicated MA concentrations. [Ribozyme] = 1 µM, [Effector] = 2 µM, [Substrate] = 0.25 µM. The reaction occurred in aqueous solution containing 100 mM NaCl, 30 mM Tris-HCl, pH 7.7, 1 mM EDTA and 2 mM Mg^2+^, and was incubated for 40 h. (**b**) Fraction of ligated substrate calculated as the band intensity of ligated substrate with MA normalized to that without fatty acid. Three independent titration experiments were performed; error bars represent standard deviations. The solid line is a sigmoidal curve fit.

Having established that stable vesicles can form at 2 mM Mg^2+^ in buffered solution compatible with L1 ligase ribozyme activity, we examined the influence of preformed vesicles at varying MA concentrations on the yield of ligation. After 40 h reaction, the amount of ligated substrate decreases as a result of increasing concentration of MA (Figure 6). An estimation of the MA concentration corresponding to half-maximal ligation efficiency compared to MA-free reaction is 10 mM. These experiments demonstrate that a suspension of fatty acid vesicles composed of 5 mM MA in an ion-rich aqueous medium including 2 mM Mg^2+^ (comprising an EDTA-bound fraction), 100 mM NaCl, pH 7.7, by retaining full ribozyme activity, offers compromising conditions for simultaneous R8-9 ligase-catalyzed reaction and MA vesicle stability.

Factors that could affect ribozyme activity under exposure to MA vesicles include Mg^2+^ depletion through fatty acid association and RNA adsorption to the vesicle membrane. The amount of free Mg^2+^ in solution is likely reduced in the presence of MA vesicles because of its association to the fraction of negatively charged fatty acids [12,14], thereby reducing ribozyme activity. On the other hand, adsorption of ribozyme to the fatty acid membrane might hamper the RNA catalytic activity through misfolding or unfavorable association with the oligonucleotide substrate. The interaction of the hammerhead ribozyme N15min7 to MA-containing vesicles has previously been studied and only a small fraction of RNA was found adsorbed to the membrane [14]. Therefore, we attribute the loss of ribozyme activity primarily to MA-based depletion of Mg^2+^, whose free concentration becomes limiting for the ligation reaction. Further experiments are required to examine R8-9 ligase adsorption to MA bilayers.

### 3.5. Low-Mg^2+^ Concentration Increases the Lifetime of Active Ligase Ribozyme

**Figure 7:**
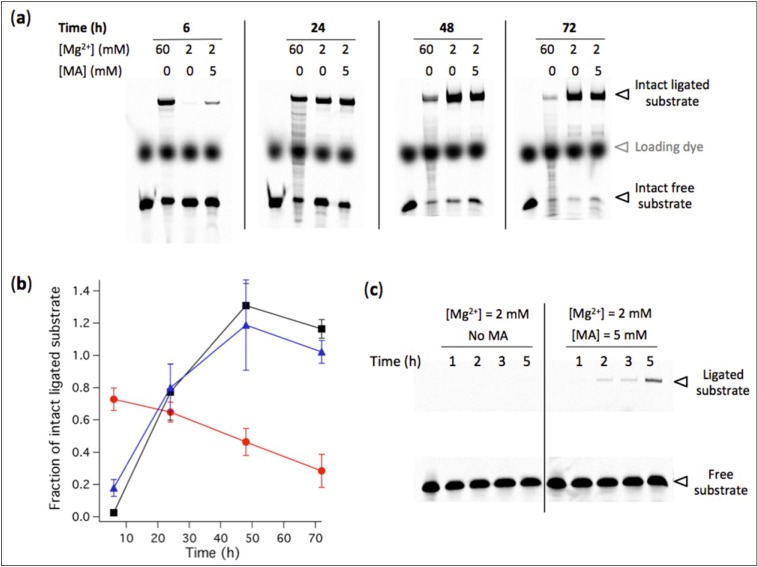
(**a**) Effects of Mg^2+^ concentration and MA vesicles on the degradation rate of RNA ligase. [Ribozyme] = 1 µM, [Effector] = 2 µM, [Substrate] = 0.25 µM. The reaction was performed at 37 ºC in aqueous medium containing 100 mM NaCl, 30 mM Tris-HCl, pH 7.7, 1 mM EDTA and the indicated concentrations of Mg^2+^ and MA. The reaction time is also indicated. Degradation bands of both ligated and free substrates are visible. In the first lane of each gel, a solution containing the substrate only was freshly added before loading the gel and was used as a reference for the total amount of substrate *S*_0_. (**b**) The fraction of intact ligated substrate was determined for each condition as the amount of intact ligated substrate measured on gel divided by *S*_0_. Red symbols, 60 mM Mg^2+^; black symbols, 2 mM Mg^2+^; Blue symbols, 2 mM Mg^2+^ and 5 mM MA vesicles. Two independent kinetics experiments were performed; error bars represent standard deviations. (**c**) Kinetics of R8-9 ribozyme-catalyzed ligation at 2 mM Mg^2+^ with or without 5 mM MA vesicles. Reaction conditions are identical as in (a), except the ligation durations.

Mg^2+^-induced RNA hydrolysis, by incapacitating ribozyme activity, is a major obstacle to a viable ribozyme-based protocell. Taking advantage of the tolerance of the R8-9 ligase ribozyme to operate at 2 mM Mg^2+^, we then asked whether the amount of intact ligated substrate could become higher under lower Mg^2+^ condition when RNA degradation becomes preponderant. We hypothesized that, despite higher ligation rate at 60 mM Mg^2+^, the RNA degradation propensity under such high concentration of Mg^2+^ ions would inactivate the ligase upon strand truncation and reduce the lifetime of substrate-ligated ribozyme. To test this possibility we quantified the amount of intact (full-length) ligated substrate at 2 mM and 60 mM Mg^2+^ after different reaction periods at 37 ºC (Figure 7). RNA fragments are seen as extra bands of smaller size below the intact ligation product and free substrate. The results show that after 24 h incubation the yield of ligated substrate becomes higher at low Mg^2+^ concentration, a consequence of the faster RNA fragmentation under high-Mg^2+^ ion condition which outcompetes the benefit of the higher ligation rate.

This finding suggests that low-Mg^2+^ conditions, by extending the lifetime of oligonucleotide substrate, active ribozyme and reaction product, provide higher evolutionary advantage.

### 3.6. Low Concentration of MA Vesicles Enhances the Kinetics of Ribozyme-Mediated Ligation

In the course of studying the effect of Mg^2+^-dependent ribozyme degradation, we observed that the presence of 5 mM MA vesicles led to larger amount of intact ligated substrate after 6 h reaction at 37 ºC under low-Mg^2+^ conditions than that in the absence of vesicles (Figure 7a,b). We decided to conduct kinetics experiments under shorter ligation durations to explore this unexpected result. Consistently, the level of ligated substrate was higher in the presence of liposomes at all measured time points (Figure 7c).

Though the mechanism underlying this vesicle-based enhancement of RNA ligase activity remains to be elucidated, plausible scenarios include an active role of the vesicle membrane [27] conferring improved stabilization of the effector-ribozyme complex or faster substrate association. A new vesicle-assisted molecular crowding mechanism can also be envisaged. This result might have some implication regarding possible coevolution of catalytic RNA and fatty acid vesicles.

## 4. Conclusions

In this study, we demonstrated that the presence of 2 mM Mg^2+^ in a buffered solution containing 1 mM EDTA, 0.1 M NaCl and MA vesicles (5 mM amphiphile) offers optimal conditions for combined L1 RNA ligase activity and lipid vesicle stability. Because higher rate of ribozyme-catalyzed reaction under high-Mg^2+^ requirement comes at the expense of lower RNA integrity, we found that the amount of intact ligation product was higher at low Mg^2+^ concentration.

Despite the importance of integrating catalytic RNA and fatty acid vesicles into the same working environment, examples of ribozyme activity in the presence of liposomes are scarce. We believe that a similar approach could be applied to explore the compatibilities of other families of ribozymes and other types of fatty acids in terms of Mg^2+^ requirement. Although other ribozymes may have lower tolerance for low concentration of Mg^2+^ than the R8-9 ligase, our results suggest that the benefit of increasing RNA lifetime under low-Mg^2+^ conditions could overcompensate the loss of activity compared to that at high concentration of divalent cations.

## References

[1] Szostak J.W., Bartel D.P., Luisi P.L. (2001). Synthesizing life. Nature.

[2] Chen I.A., Walde P. (2010). From self-assembled vesicles to protocells. Cold Spring Harb. Perspect. Biol..

[3] Attwater J., Hollinger P. (2014). A synthetic approach to abiogenesis. Nat. Methods.

[4] Walde P., Wick R., Fresta M., Mangone A., Luisi P.L. (1994). Autopoietic self-reproduction of fatty acid vesicles. J. Am. Chem. Soc..

[5] Hanczyc M.M., Fujikawa S.M., Szostak J.W. (2003). Experimental models of primitive cellular compartments: Encapsulation, growth and division. Science.

[6] Zhu T.F., Szostak J.W. (2009). Coupled growth and division of model protocol membranes. J. Am. Chem. Soc..

[7] Zhang S., Blain J.C., Zielinska D., Gryaznov S.M., Szostak J.W. (2013). Fast and accurate nonenzymatic copying of an RNA-like synthetic genetic polymer. Proc. Natl. Acad. Sci..

[8] Attwater J., Wochner A., Holliger P. (2013). In-ice evolution of RNA polymerase ribozyme activity. Nat. Chem..

[9] Misra V.K., Draper D.E. (2002). The linkage between magnesium binding and RNA folding. J. Mol. Biol..

[10] Bowman J.C., Lenz T.K., Hud N.V., Williams L.D. (2012). Cations in charge: Magnesium ions in RNA folding and catalysis. Curr. Opin. Struct. Biol..

[11] Szostak J.W. (2012). The eightfold path to non-enzymatic RNA replication. J. Syst. Chem..

[12] Monnard P.-A., Apel C.L., Kanavarioti A., Deamer D.W. (2002). Influence of ionic inorganic solutes on self-assembly and polymerization process related to early forms of life: implication for a prebiotic aqueous medium. Astrobiology.

[13] Apel C.L., Deamer D.W., Mautner M.N. (2002). Self-assembled vesicles of monocarboxylic acids and alcohols: Conditions for stability and for the encapsulation of biopolymers. Biochimica et Biophysica Acta.

[14] Chen I.A., Salehi-Ashtiani K., Szostak J.W. (2005). RNA catalysis in model protocell vesicles. J. Am. Chem. Soc..

[15] Mansy S.S., Szostak J.W. (2008). Thermostability of model protocell membranes. Proc. Natl. Acad. Sci..

[16] Adamala K., Szostak J.W. (2013). Nonenzymatic template-directed RNA synthesis inside model protocells. Science.

[17] Athavale S.S., Petrov A.S., Hsiao C., Watkins D., Prickett C.D., Gossett J.J., Lie L., Bowman J.C., O’Neill E., Bernier C.R., Hud N.V., Wartell R.M., Harvey S.C., Williams L.D. (2012). RNA folding and catalysis mediated by iron (II). PLoS One.

[18] Robertson M.P., Ellington E. (1999). *In vitro* selection of an allosteric ribozyme that transduces analytes to amplicons. Nat. Biotech..

[19] Robertson M.P., Scott W.G. (2007). The structural basis of ribozyme-catalyzed RNA assembly. Science.

[20] Robertson M.P., Hesselberth J.R., Ellington A.D. (2001). Optimization and optimality of a short ribozyme ligase that joins non-Watson-Crick base pairings. RNA.

[21] Leach W.W., Nooner D.W., Oro J., Noda H. (1978). Origins of Life.

[22] Deamer D.W. (1985). Boundary structures are formed by organic components of the Murchison carbonaceous chondrite. Nature.

[23] Mautner M.N., Leonard R.L., Deamer D.W. (1995). Meteorite organics in planetary environments: Hydrothermal release, surface-activity, and microbial utilization. Planet Space Sci..

[24] Gilbert W. (1986). Origin of life: The RNA world. Nature.

[25] Segré D., Ben-Eli D., Deamer D.W., Lancet D. (2001). The lipid world. Orig. Life Evol. Biosph..

[26] Schneider C.A., Rasband W.S., Eliceiri K.W. (2012). NIH Image to ImageJ: 25 years of image analysis. Nat. Methods.

[27] Walde P., Umakoshi H., Stano P., Mavelli F. (2014). Emergent properties arising from the assembly of amphiphiles. Artificial vesicle membranes as reaction promoters and regulators. Chem. Commun..

